# Hypohidrotic ectodermal dysplasia syndrome in a Sudanese patient: a case report

**DOI:** 10.11604/pamj.2025.52.133.48575

**Published:** 2025-11-28

**Authors:** Mohamed Abdullatif Mukhtar, Ahmed Alnazear Abdalla Amine, Sahar Abdelatif Muctar, Muntasir Eltayeb Ibrahim, Hiba Salaheldin Mohamed

**Affiliations:** 1 Institute of Endemic Diseases, University of Khartoum, Khartoum, Sudan,; 2 Department of Microbiology, University of California, San Francisco, CA 94143, USA,; 3 The G.W. Hooper Foundation, San Francisco, CA 94143, USA,; 4 Department of Periodontology, Faculty of Dentistry, Military Dental Hospital, Karary University, Omdurman, Sudan,; 5 Department of Biology, College of Science, Taibah University, Medina, Saudi Arabia

**Keywords:** Ectodermal dysplasia, EDA, X-linked hypohidrotic ectodermal dysplasia, ectodysplasin-A, case report

## Abstract

Hypohidrotic ectodermal dysplasia (HED) is a rare genodermatosis characterised by defects in the development of ectodermal tissues, primarily affecting hair, teeth, and sweat glands. It can be inherited in autosomal dominant, autosomal recessive, or X-linked patterns. Mutations in the EDA, EDAR, or EDARADD genes, which encode Ectodysplasin-A, the EDA receptor, and a death domain, respectively, are responsible for HED. A 36-year-old Sudanese male presented with hypohidrosis, hypodontia, hypotrichosis, and Ichthyosis without an obvious family history of the HED syndrome. Six members of his extended family were selected to investigate the mutation in the EDA gene and its inheritance. No deletions or mutations were detected in EDA coding exons. The patient is advised on environmental modifications to avoid overheating. This case highlights the potential involvement of other genes and recommends further genetic testing.

## Introduction

Ectodermal dysplasia syndrome (ED) is a rare, diverse group of disorders involving developmental defects of two or more ectodermal structures [1]. One of these types is Hypohidrotic ectodermal dysplasia (HED), which is inherited in an X-linked (XL), autosomal recessive (AR), or autosomal dominant (AD) manner. In some cases, they can occur in individuals without a family history, this is referred to as a de novo mutation [2]. The most prevalent form is XL-HED (OMIM #305100), which affects 1-10 out of every 100,000 live male births. The carrier incidence is around 17.3 in 100,000 women, accounting for 95% of cases of Ectodermal Dysplasias [3]. XL-HED is caused by mutations in the EDA gene (locus Xq12-q13), which encodes ectodysplasin-A, a type II transmembrane protein of 391 amino acids [2]. Autosomal recessive-HED (OMIM #224900) and Autosomal dominant-HED (OMIM #129490) are caused by mutations in the EDA receptor (EDAR chromosome 2q13), or the cytosolic EDAR-specific adaptor molecule called EDAR-associated death domain (EDARADD chromosome 1q42-q43), respectively [2].

It is difficult to differentiate between these three forms clinically, most likely because they alter a single signal transduction pathway. Certainly, the binding of ectodysplasin to its receptor, EDAR, allows the recruitment of EDARADD as an adapter to activate the NF-κB signaling pathway. EDA affects the development of hair, teeth, and several exocrine glands, including sweat, meibomian, and preputial glands. This pathway is necessary for the initiation, formation, and differentiation of skin appendages [4]. The clinical features of XL-HED include hypohidrosis (or anhidrosis), sparse hair and eyebrows (atrichosis or hypotrichosis), periorbital wrinkling, dry skin, abnormal and missing teeth (anodontia or hypodontia), frequent incidence of respiratory infections, as well as hypoplasia of sweat, sebaceous, meibomian, mammary, and lachrymal glands [4]. We report a case of hypohidrotic ectodermal dysplasia in a Sudanese male who underwent genetic testing.

## Patient and observation

**Figure 1:**
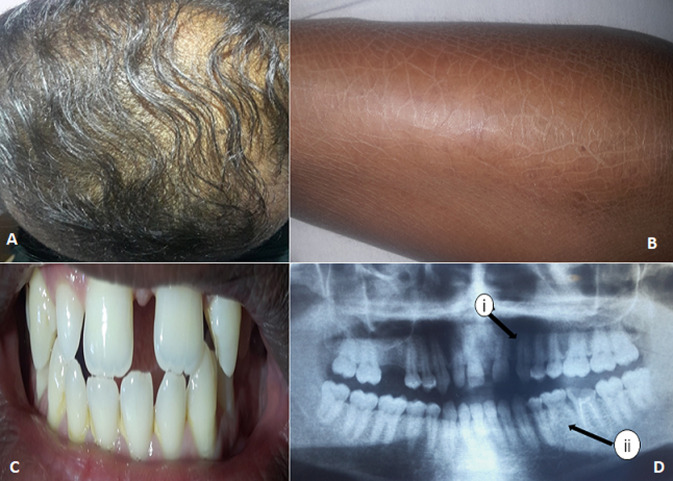
clinical observations of the patient: A) sparse scalp hair; B) ichthyosis; C) missing deciduous upper left lateral incisor; D) (i) missing deciduous upper left lateral incisor; (ii) primary second molar

**Patient information:** the proband was a 36-year-old male with mild typical traits of HED, including hypohidrosis, hypotrichosis, and hypodontia. He reported recurrent episodes of hyperthermia, especially in hot weather. He had light and dry scalp hair (Figure 1A), prominent ears, and protruding lips. Ichthyosis has also been detected on the legs (Figure 1B), as well as dry skin and sole hyperkeratosis.

**Timeline of current episode:** the patient has had persistent symptoms since birth, including features consistent with ectodermal dysplasia. In 2012, during a dental evaluation, clinical suspicion of Hypohidrotic ectodermal dysplasia was raised. This led to referral to a specialized genetic center, where molecular testing was performed in 2014.

**Clinical findings:** intraoral examination showed a missing deciduous upper left lateral incisor (Figure 1C, D), presence of primary second molar, and absence of permanent successor (second premolar) of orthopantomogram (Figure 1D). However, his mental and physical development was normal, and no abnormalities in his finger and toenails were identified.

**Figure 2:**
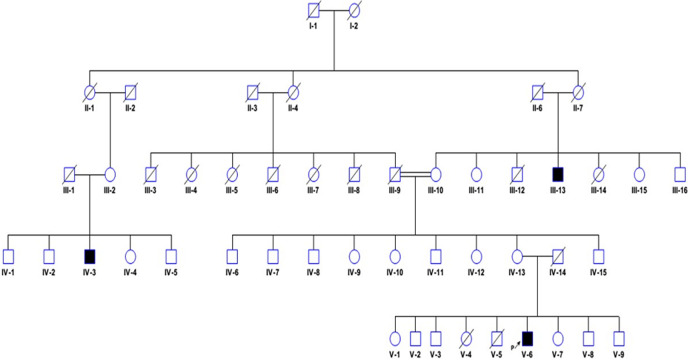
pedigree of the family analyzed: the proband (V-6) is marked with an arrow; filled symbols represent the suspected affected family members according to clinical investigations

**Figure 3:**
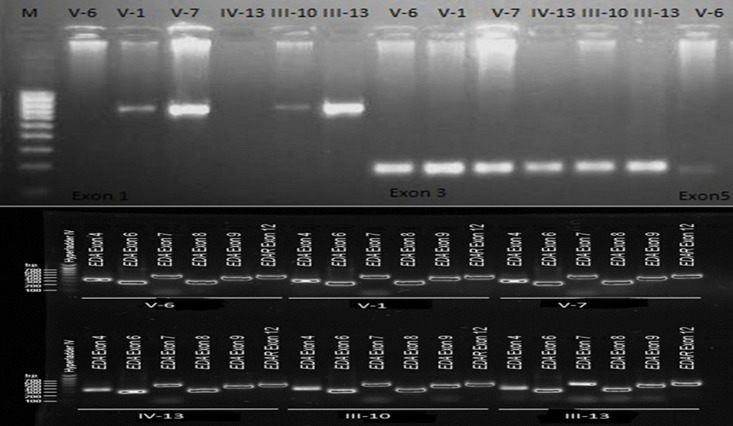
electrophoresis result of the amplified regions: A) electrophoresis result of the amplified regions for EDA gene Exon 1,3 and 5; B) electrophoresis result for EDA amplified regions for exon 4,6,7,8 9 and EDAR exon 12 as internal control

**Diagnostic assessment:** blood samples were taken from the proband and the other five family members (V-1, V-7, IV-13, III-10, and III-13) (Figure 2) to confirm the maternal or de novo origin of the mutation. DNA was isolated using the standard Guanidine method. Polymerase chain reaction (PCR) fragments of 240-732bp, corresponding to exons 1-9, were amplified using primers demonstrated in Table 1. In brief, 30ng of DNA was amplified by PCR in a 50µl PCR mix containing: 1X buffer, 0.4mM dNTPs, 2mM MgCl2, 0.4pmol of each primer, and 1U Taq polymerase. Following an initial DNA denaturation step of 95°C for 3 min, 40 cycles of amplification were performed using the following cycling parameters: denaturation at 95°C for 30 s; and multistep annealing at 56°C -65°C for 30s for 1.5 min. A final extension at 72°C for 10 min followed the last cycle. The amplified exons were sequenced in two sets, exons (4, 6, 7, 8, and 9) at Roslin Institute and Royal (Dick) School of Veterinary Studies and the other (exons 1, 3, and 5) were sequenced commercially at Macrogene company (Korea), and analyzed using BioEdit sequence alignment editor version 7.2 software at the Institute of Endemic Diseases. Exon 2 was omitted since no mutations have been identified in previous research. The sequences were compared to the EDA reference sequence in the ensemble database. All participants showed clear bands for each exon in the polymerase chain reaction (Figure 3). No mutation was detected after the sequencing of the 8 exons and the nucleotide sequence of the amplified fragments of these regions from the proband, the other five examined family members, and the reference sequence showed complete accordance.

**Table 1:** primers used for polymerase chain reaction (PCR) amplification of the ectodysplasin-A (EDA) gene

Product Length	Length	Sequence 5'→3'	Exon
EDA-EX-1	F: CTCGGAGTAGAGCTGCACAT	20	732[8]
	R: TGGGCAACTTCTCCCTTGCT	20	
EDA-EX-3	F: TTATGTTGGCTATGACTGAGTGG	23	258[9]
	R: CCACCATGCCCTACCAAGAAGGT	23	
EDA-EX-4	F: TTTGCAGTGTCTTGGGGATCC	21	347[2]
	R: GCAGGGAGAAGAACAAGGAAGAAT	24	
EDA-EX-5	F: AAAAAAGTAACACTGAATCCTATT	24	240[9]
	R: CTGGTGGGGTGGAGAGACAG	20	
EDA-EX-6	F: GAATAAAGCTCAGACAGGGC	20	273[10]
	R: AATCTCCGGGGTGTTCTCAT	20	
EDA-EX-7	F: CATAGCTAGGAAGCGGTAGA	20	474[8]
	R: CTCCGTCATCAGTGATTCTA	20	
EDA-EX-8	F: TGCCTCGATTATTCTGACATGTACTG	26	300[2]
	R: CCCAAAGCAGGAAGTTAGCCATT	23	
EDA-EX-9	F: CCCCACCCTCTCTTTCCTCTCTTC	24	413[2]
	R: GGCTGCAACACCAATACACCTCAC	24	

**Diagnosis:** the patient was clinically diagnosed as hypohidrotic ectodermal dysplasia with no EDA gene mutation.

**Therapeutic interventions:** no specific therapy was provided as it has no approved clinically effective treatment for such a disease. The patient was advised on behavioral and environmental amendments to avoid overheating and referred to genetic counseling.

**Follow-up and outcome of interventions:** no complications or progression reported post-evaluation. The patient remained stable with avoidance of thermal triggers, hydration, and recurrent skin moisturization.

**Patient perspective:** the patient was aware of the study objectives and motivated to participate to contribute to the scientific understanding of this condition.

**Informed consent:** the patient is deceased, so informed and signed consent was obtained from the patient's caregiver.

## Discussion

HED is characterized by different degrees of abnormalities of teeth, sparse hair, and absent or reduced sweating [1]. When an affected male represents a HED case without a clear family history, the possibilities include either he or his mother having a de novo disease-causing mutation in the EDA gene. When his mother carries a de novo mutation, it could be either as a germline mutation or as germline mosaicism; the third possibility his grandmother has a de novo disease-causing mutation [5].

According to the clinical investigations in this family, we presumed that this mutation could be of an X-linked pattern, but the symptoms in the suspected patients are extremely mild, and the imprecise clinical history for most of the family members prevents us from ruling out a de novo mutation. We selected the participants in this study to cover all these possibilities of X-linked or sporadic mutation, and we included the suspected affected and carriers. One of the suspected patients (IV-3) refused to participate in the study.

Previous studies have shown that the detection rates of EDA mutations range from 65% to 94%. Moreover, about 80% of the EDA mutations identified in XL-HED subjects are small intragenic changes, including point mutations, small deletions, and insertions. As well, large deletions, even loss of an entire exon and complete gene deletion, have also been found [6].

Mutations in the transmembrane domain, encoded by exon 1, interfere with transmembrane transport and possibly change the polarity of amino acids. Mutations in the collagen domain encoded by exons 5 and 6 can inhibit multimerization of the TNF homology region, while those in the consensus furin recognition sequence, which is encoded by exon 3, prevent proteolytic cleavage of EDA. The mutations in exons 7-9 in the TNF homology domain will impair the specific binding of both EDA splice variants to their receptors. Some researchers have suggested that 98% of EDA mutations are located in exons 1, 3, 5, 8, and 9. Further, the CpG-containing arginine codon at position 156 in exon 3 is considered a mutation hot spot in the EDA gene [6-10].

## Conclusion

There are no EDA gene mutations or deletions found in this patient. Furthermore, because HED is not thought to have a strong genotype/phenotype correlation [6], inter-familial differences in expressivity in individuals with the same mutation, as well as intra-familial variability in affected brothers, have been noticed [6,7]. We believe that the disease-causing mutation may be in one of the other causative genes, and exome sequencing is recommended.
